# The influence of adolescents' perceived task value in sport on exercise adherence: the chain mediating roles of general self-efficacy and action planning

**DOI:** 10.3389/fpsyg.2026.1828334

**Published:** 2026-05-28

**Authors:** Jingwen Zhang, Zhenyu Wang, Yang Zhou, Wen Jing, Pengwei Song

**Affiliations:** 1 School of Physical Education and Health, Guangxi Science and Technology Normal University, Laibin, Guangxi, China; 2 Keimyung University, Daegu, Republic of Korea; 3 School of Physical Education, Bohai University, Liaoning, Liaoning, China

**Keywords:** action planning, adolescents, exercise adherence, general self-efficacy, perceived task value in sport

## Abstract

**Objective:**

This study examined the association between adolescents' perceived task value in sport and exercise adherence, as well as whether this association was explained by a serial mediation pathway through general self-efficacy and action planning.

**Methods:**

A questionnaire-based cross-sectional survey was conducted from September 15 to December 15, 2025. Participants were 572 Chinese secondary-school students recruited from Tianjin, Guangxi, Hebei, and Liaoning. Measures assessed perceived task value in sport, general self-efficacy, action planning, and exercise adherence. Pearson correlations were computed, and a serial mediation model was tested using SPSS PROCESS (Model 6) with standardized coefficients and 5,000 bootstrap resamples to generate bias-corrected 95% confidence intervals (CIs).

**Results:**

Perceived task value in sport was positively correlated with general self-efficacy (*r* = 0.184), action planning (*r* = 0.238), and exercise adherence (*r* = 0.372; all *p* < 0.001). General self-efficacy correlated with action planning (*r* = 0.324) and exercise adherence (*r* = 0.321), and action planning correlated with exercise adherence (*r* = 0.291) (all *p* < 0.001). Perceived task value predicted general self-efficacy (β = 0.184, *p* < 0.001) and, with general self-efficacy, predicted action planning (β = 0.185 and β = 0.290, respectively; both *p* < 0.001). In the final model, all three predicted exercise adherence (β = 0.296, β = 0.218, and β = 0.150, respectively; all *p* < 0.001). Bootstrap analyzes supported partial mediation: the total effect was β = 0.372 (95% BootCI [0.296, 0.449]) and the direct effect remained significant at β = 0.296 (95% BootCI [0.221, 0.372]). All indirect pathways were significant: the general self-efficacy path (0.040, 95% BootCI [0.019, 0.067]), the action planning path (0.028, 95% BootCI [0.010, 0.052]), and the serial path via general self-efficacy and action planning (0.008, 95% BootCI [0.003, 0.016]); the total indirect effect was 0.076 (95% BootCI [0.044, 0.114]).

**Conclusion:**

Adolescents who perceive higher task value in sport report stronger exercise adherence, both directly and indirectly through greater general self-efficacy and more concrete action planning. Interventions may enhance adherence by strengthening task value perceptions, building efficacy through mastery experiences and feedback, and training adolescents to formulate feasible action plans.

## Introduction

Insufficient physical activity and the high prevalence of sedentary behavior among adolescents have become major public health concerns that threaten their physical and mental health (Bueno-Antequera and Munguía-Izquierdo, 2023). A long-term lack of regular exercise not only increases physiological risks such as overweight/obesity and reduced cardiorespiratory fitness, but is also closely associated with psychological problems including anxiety and depression, as well as poor sleep quality (Chrysant and Chrysant, 2023; Xiang et al., 2021). Across both school physical education and leisure-time contexts, whether adolescents' sport participation can be translated into sustained, regular behavioral habits is central to improving physical fitness and fostering lifelong engagement in physical activity (Green and Johansen, 2025).

Perceived task value in sport refers to adolescents' subjective cognition and evaluation of the meaning and benefits of sport participation—essentially an overall judgment that “sport is valuable and beneficial to me” (Stuart, 2003). Self-determination theory (SDT) posits that recognizing and internalizing the value of a behavior provides a key psychological basis for sustained engagement (Coccia, 2019). However, an “intention–action” gap is common in adolescents' physical activity participation (Feil et al., 2023): even when adolescents endorse the value of sport and form an intention to exercise, they may still be constrained by academic pressure, peer influence, and situational barriers, making it difficult to translate intention into long-term adherence (Zhang et al., 2022). Therefore, it is necessary to clarify the behavioral change mechanisms through which perceived task value, after intentions have formed, is converted into the formation and maintenance of exercise adherence via key psychological processes.

Exercise adherence refers to an individual's sustained compliance with a prescribed exercise plan and the consistency of behavior implementation, typically involving stable execution of exercise frequency, intensity, and duration. It is a core indicator for adolescents to develop healthy exercise habits and to achieve health benefits through physical activity (Zou et al., 2023). Prior research has shown a significant positive association between perceived task value in sport and exercise adherence, suggesting that a more positive valuation of sport tasks can enhance the sustainability of exercise behavior (Barbosa Cano and Gómez-Baya, 2025). Nevertheless, empirical evidence that systematically tests the “perceived task value–exercise adherence” pathway and its underlying mechanisms specifically among adolescents remains limited.

The Health Action Process Approach (HAPA) provides a clear theoretical framework for explaining how exercise intentions are translated into stable exercise behavior among adolescents. Intentions do not necessarily lead to actual behavior; beyond the motivational phase, self-efficacy and action planning are considered core mechanisms that support sustained action. Self-efficacy reflects adolescents' beliefs in their capability to complete exercise tasks and overcome barriers, whereas action planning helps transform abstract intentions into operational steps and coping strategies, thereby supporting the frequency, intensity, and continuity of exercise and ultimately promoting the formation and maintenance of exercise adherence (Renner and Schwarzer, 2009). Compared with sport-specific self-efficacy, general self-efficacy—as a cross-situational and relatively stable psychological resource—may better reflect adolescents' confidence in coping with multiple barriers such as academic demands and time conflicts. In the present adolescent context, general self-efficacy may be particularly relevant because exercise adherence is often challenged by barriers beyond sport itself, such as academic pressure, time conflicts, and changing daily schedules. Following the core logic of HAPA, it is reasonable to infer that perceived task value in sport may first enhance general self-efficacy, which in turn facilitates the development of clear and feasible action plans, thereby improving exercise adherence. This implies that perceived task value may have a direct effect on exercise adherence and may also exert an indirect effect through a chain pathway of “self-efficacy → action planning.” However, direct empirical support for this chain mediation mechanism in adolescent samples is still lacking.

Accordingly, the present study aims to examine a chain mediation model involving perceived task value in sport, general self-efficacy, action planning, and exercise adherence among adolescents. Drawing on the Health Action Process Approach and expectancy-value theory, this study further examines whether general self-efficacy and action planning sequentially mediate the association between perceived task value in sport and exercise adherence. This model may help clarify the psychological and planning processes associated with adolescents' exercise adherence and provide empirical evidence for school-based physical activity promotion.

## Literature review and research hypotheses

### Perceived task value in sport and exercise adherence

Perceived task value in sport refers to an individual's overall judgment of whether engaging in physical exercise is “worth the investment.” It typically encompasses dimensions such as importance, enjoyment, and usefulness, and reflects a comprehensive evaluation of expected outcomes and participation experiences (Stuart, 2003). According to expectancy–value theory, value appraisals of a given behavior directly influence individuals' willingness to invest, the effort they exert, and the extent to which they persist in that behavior (Feather, 1992). In the context of sport participation, adolescents who regard exercise as important, beneficial, or enjoyable are more likely to prioritize it in their time allocation and behavioral choices, and to maintain higher levels of persistence when they are faced with fatigue or short-term difficulties (Zhang et al., 2022). From the perspective of self-determination theory, perceived task value reflects a process of recognizing and internalizing the meaning of exercise: when exercise shifts from an “external requirement” to a “personally endorsed and meaningful behavior,” motivation becomes more stable, thereby facilitating the sustained enactment of exercise behavior (García-Calvo et al., 2011). Existing research has supported a positive association between perceived task value in sport and exercise adherence among adolescents. However, much of the evidence remains correlational, and the internal mechanisms through which task value specifically influences adherence have not been sufficiently examined (Gu et al., 2014). Taken together, theoretical accounts and prior findings suggest that perceived task value in sport is likely to directly and positively predict adolescents' exercise adherence by shaping a stable and favorable behavioral tendency.

Accordingly, we proposed Hypothesis 1 (H1): Perceived task value in sport positively predicts adolescents' exercise adherence.

### The mediating role of general self-efficacy

General self-efficacy refers to an individual's overall belief in their capability to successfully cope with challenges and achieve goals (Sánchez and Guo, 2023). In adolescents' engagement in physical exercise, it represents a key psychological resource that helps individuals overcome difficulties and maintain long-term participation (Hagger et al., 2001). According to social cognitive theory, positive value appraisals of a behavior constitute an important prerequisite for enhancing self-efficacy (Abdullah, 2019). When adolescents endorse the value of sport tasks, they are more likely to participate in exercise proactively and persistently, thereby accumulating mastery experiences and receiving positive feedback during the process (Yli-Piipari et al., 2013). Such direct experiences of success are a core source for developing and consolidating a general capability belief that “I can maintain exercise.” Accordingly, perceived task value in sport is expected to positively predict general self-efficacy. In turn, general self-efficacy has been shown to directly promote exercise adherence (Li et al., 2025; Zhang et al., 2024). Within the Health Action Process Approach (HAPA), adolescents with higher self-efficacy tend to demonstrate stronger persistence and psychological resilience when encountering common barriers such as fatigue, time conflicts, or motivational fluctuations (Renner and Schwarzer, 2009). They are more likely to interpret difficulties as manageable challenges and to adopt active problem-solving strategies rather than giving up, which is reflected in higher adherence at the behavioral level. Taken together, perceived task value in sport may enhance adolescents' exercise adherence by strengthening general self-efficacy, thereby improving their capacity to implement exercise plans in the face of barriers.

Accordingly, we proposed Hypothesis 2 (H2): General self-efficacy mediates the relationship between perceived task value in sport and adolescents' exercise adherence.

### The mediating role of action planning

Action planning refers to the concrete arrangements individuals formulate regarding when, where, and how to engage in exercise after forming an exercise intention, and it constitutes a critical step in translating intention into actual behavior (Rebar et al., 2023). Research indicates that behavioral intention alone is insufficient to ensure behavior enactment; stable implementation largely depends on the formulation of clear, specific, and actionable plans (Ajzen, 1985). The Health Action Process Approach (HAPA) further emphasizes that action planning serves as a core self-regulation strategy in the transition from intention to action. By specifying implementation details, it can reduce uncertainty during execution and alleviate the burden of in-the-moment decision-making, thereby increasing the likelihood of behavior initiation and persistence (Renner and Schwarzer, 2009).

Adolescents' exercise arrangements are often disrupted by academic demands, daily routines, and emotional fluctuations. Without concrete plans, exercise is easily replaced by other *ad hoc* activities; in contrast, a clear and feasible plan facilitates the integration of exercise into daily life and promotes the development of stable behavioral routines (Derhemi and Kovaci, 2025). From a value-based perspective, individuals with higher perceived task value in sport are more willing to invest cognitive resources in planning future behaviors, thereby being more likely to develop high-quality action plans. In turn, such specific plans can translate abstract value endorsement into a clear, actionable behavioral pathway, guiding individuals to implement their exercise arrangements and ultimately exhibit higher levels of exercise adherence.

Accordingly, we proposed Hypothesis 3 (H3): Action planning mediates the relationship between perceived task value in sport and adolescents' exercise adherence.

### The chain mediating roles of general self-efficacy and action planning

Within the theoretical framework of the Health Action Process Approach (HAPA), self-efficacy and action planning jointly constitute core self-regulatory mechanisms that promote behavior initiation and maintenance during the intention–action phase (Renner and Schwarzer, 2009). Conceptually, efficacy beliefs often precede the formation of concrete behavioral strategies: general self-efficacy, as a key psychological resource, supports individuals in coping with barriers and pursuing behavioral goals, whereas action planning further translates these beliefs into clear and executable behavioral pathways. In the present study, perceived task value in sport, as a cognitive antecedent, may first enhance adolescents' general self-efficacy by increasing their confidence in coordinating time, overcoming fatigue, and managing external interference. Subsequently, higher self-efficacy may motivate adolescents to develop specific and feasible action plans, thereby transforming exercise intentions into clear implementation arrangements. By specifying the time, location, and manner of exercise, action planning can reduce uncertainty and the burden of immediate decision-making during execution, increasing the likelihood of behavior initiation and sustained enactment and ultimately resulting in higher exercise adherence. This process reflects a progressive pathway of “value cognition → efficacy beliefs → plan enactment,” consistent with HAPA's assumption that behavior change shifts from cognitive resources toward behavioral implementation.

Accordingly, we proposed Hypothesis 4 (H4): General self-efficacy and action planning play a chain mediating role in the relationship between perceived task value in sport and adolescents' exercise adherence.

**Figure 1:**
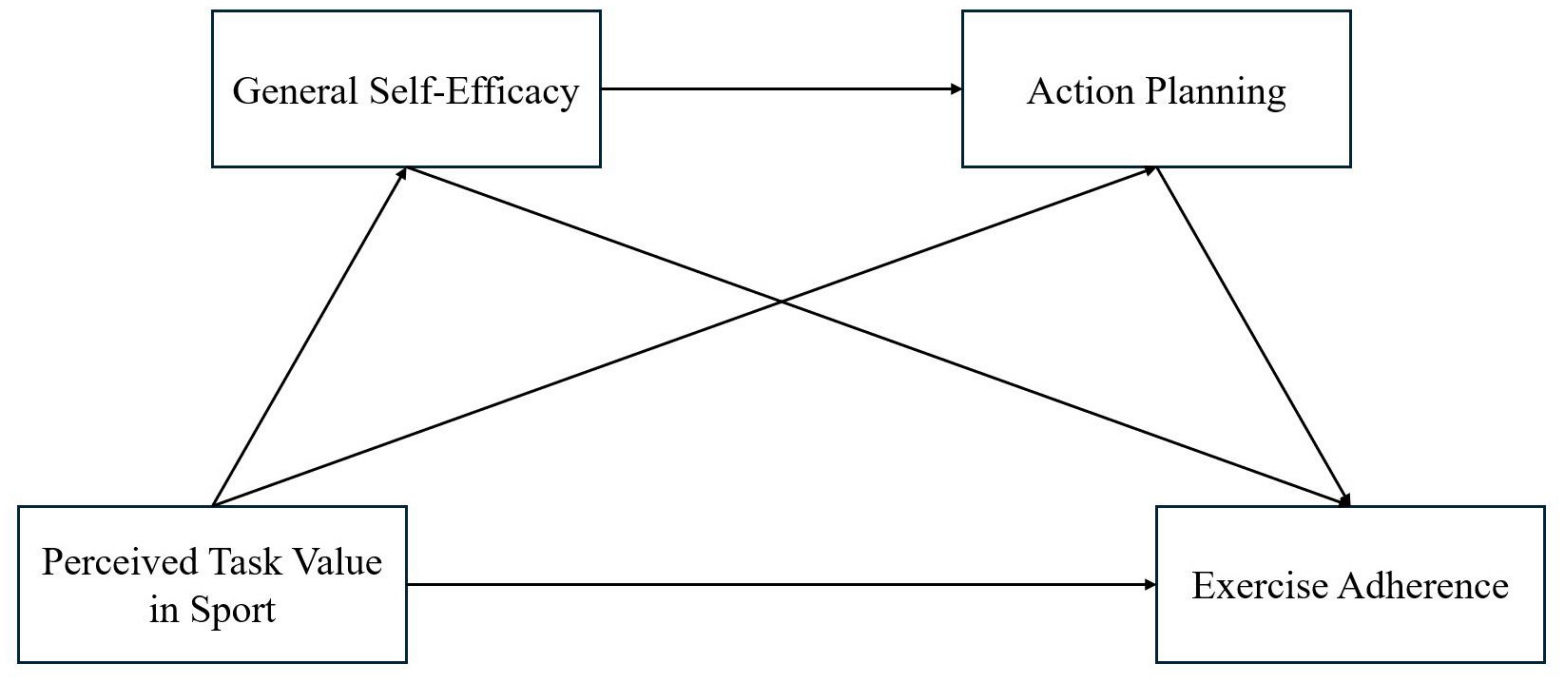
Chain mediation model.

In summary, drawing on HAPA, self-determination theory, and expectancy–value theory, the present study conceptualizes general self-efficacy and action planning as core psychological mechanisms through which adolescents' value perceptions are translated into stable exercise behavior. We therefore constructed a chain mediation model of “perceived task value in sport → general self-efficacy → action planning → exercise adherence” (Figure 1).

## Participants and methods

### Participants

This study employed a questionnaire-based survey conducted between September 15 and December 15, 2025. Participants were enrolled from multiple secondary schools in Tianjin Municipality, the Guangxi Zhuang Autonomous Region, Hebei Province, and Liaoning Province, China. A total of 650 questionnaires were collected. After validity screening, 572 were retained as valid, yielding an effective response rate of 88%. This study was approved by the Ethics Committee of the School of Physical Education and Health Sciences, Guangxi Science and Technology Normal University. Written informed consent was obtained from all participants.

### Research instruments

Four scales were used to assess adolescents' perceived task value in sport, general self-efficacy, action planning, and exercise adherence. All scales used Likert-type rating formats, and higher scores indicate higher levels of the corresponding construct. Only the Exercise Adherence Scale was reverse-coded, such that higher scores reflected greater exercise adherence.

#### Perceived task value in sport scale

The Perceived Task Value in Sport Scale developed by Østerlie et al. (2019) was used. The scale contains 6 items covering three dimensions: importance, enjoyment, and usefulness. Items are rated on a 5-point scale ranging from 1 (strongly disagree) to 5 (strongly agree), with higher scores indicating stronger perceived task value in sport. In this study, the scale showed good internal consistency (Cronbach's α = 0.868).

#### General self-efficacy scale

General self-efficacy was assessed using the scale revised by Wang et al. (2001). The scale includes 10 items rated on a 4-point scale from 1 (not at all true) to 4 (completely true), with higher scores indicating higher general self-efficacy. In this study, internal consistency was excellent (Cronbach's α = 0.935).

#### Action planning scale

Action planning was measured using the Chinese version of the Exercise Planning Scale revised by Zhu et al. (2022). The scale consists of 5 items focusing on the specificity of exercise planning. Responses are recorded on a 4-point scale from 1 (strongly disagree) to 4 (strongly agree), with higher scores indicating stronger action planning. In this study, the scale demonstrated good reliability (Cronbach's α = 0.859).

#### Exercise adherence scale

Exercise adherence was assessed using the Exercise Adherence Rating Scale developed by Newman-Beinart et al. (2017). The scale includes six core items focusing on the extent to which individuals implement and maintain a prescribed exercise plan. Items are rated on a 5-point scale from 0 (strongly agree) to 4 (strongly disagree). All items were reverse-coded so that higher scores represented higher levels of exercise adherence. In this study, the scale demonstrated good reliability (Cronbach's α = 0.894).

### Data analysis

Data were analyzed using SPSS 29.0 and the PROCESS macro (Model 6). The analyzes included tests for common method bias, reliability and validity, descriptive statistics, Pearson correlation analyzes, and regression analyzes. Bootstrap mediation procedures were used to construct models examining the relationships among perceived task value in sport, general self-efficacy, action planning, and exercise adherence, and to test both the mediation model and the chain mediation model. The significance level was set at α = 0.05.

## Results

### Test for common method bias

Common method bias was assessed using Harman's single-factor test. All 27 items from the four scales were entered simultaneously into an unrotated exploratory factor analysis. The results yielded four factors with eigenvalues greater than 1. The first factor accounted for 31.20% of the total variance, which did not exceed the commonly used 40% threshold, suggesting that common method bias was not a serious concern in this study.

### Descriptive statistics and correlation analysis

Descriptive statistics and Pearson correlation coefficients for all variables are presented in Table 1. The results indicated that perceived task value in sport was significantly positively correlated with general self-efficacy (*r* = 0.184, *p* < 0.001), action planning (*r* = 0.238, *p* < 0.001), and exercise adherence (*r* = 0.372, *p* < 0.001). General self-efficacy was also positively correlated with action planning (*r* = 0.324, *p* < 0.001) and exercise adherence (*r* = 0.321, *p* < 0.001). In addition, action planning was positively correlated with exercise adherence (*r* = 0.291, *p* < 0.001). The directions of these associations were consistent with the theoretical hypotheses, thereby providing a statistical foundation for subsequent analyzes of the chain mediation effects.

**Table 1:** Descriptive statistics and correlation analysis (*M* ± SD).

Variable	Mean	SD	1	2	3	4
Perceived task value in sport	4.40	0.55	1			
General self-efficacy	3.02	0.57	0.184^***^	1		
Action planning	2.78	0.55	0.238^***^	0.324^***^	1	
Exercise adherence	3.33	0.53	0.372^***^	0.321^***^	0.291^***^	1

^*^p < 0.05, ^**^p < 0.01, ^***^p < 0.001.

### Hypothesis testing

The chain mediation effect between perceived task value in sport and exercise adherence was examined using the PROCESS macro in SPSS 29.0. The significance of indirect effects was tested via a bootstrap procedure with 5,000 resamples and 95% confidence intervals (CIs). An indirect effect was deemed significant if the bootstrap CI did not contain zero.

**Figure 2:**
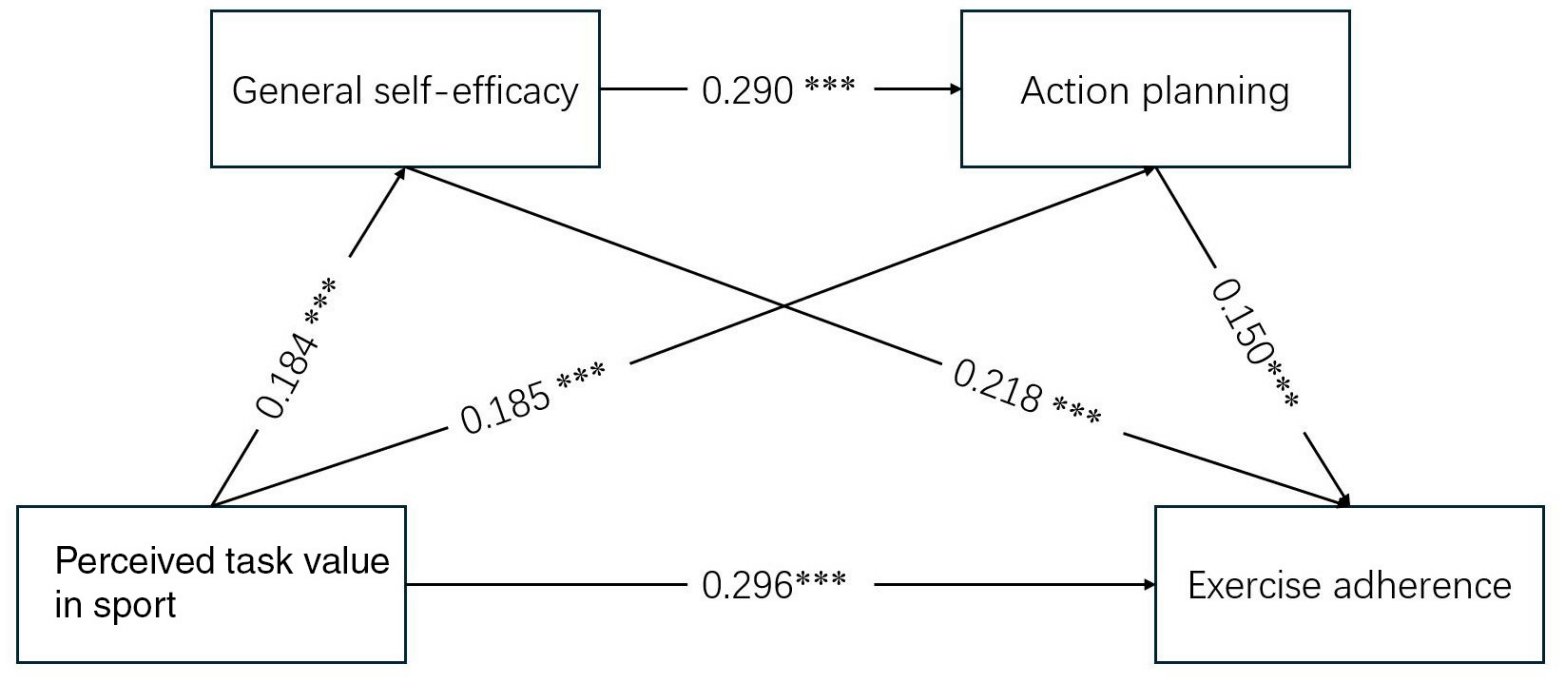
Serial mediation model. Path coefficients are standardized coefficients (β) obtained from the SPSS PROCESS output (consistent with Table 2). ^***^p < 0.001.

Regression results are presented in Table 2. Perceived task value in sport significantly positively predicted general self-efficacy (β = 0.184, *p* < 0.001). In the regression model with action planning as the dependent variable, both perceived task value in sport (β = 0.185, *p* < 0.001) and general self-efficacy (β = 0.290, *p* < 0.001) were significant positive predictors. Further analyses revealed that when perceived task value in sport, general self-efficacy, and action planning were entered simultaneously, all three variables significantly positively predicted exercise adherence (β = 0.296, *p* < 0.001; β = 0.218, *p* < 0.001; β = 0.150, *p* < 0.001, respectively). The serial mediation model is shown in Figure 2.

**Table 2:** Regression coefficients for the serial mediation model.

Regression models		Model fit statistics			Coefficient		Confidence interval	
Dependent variable	Independent variable	*R*	*R2*	*F*	β	*t*	LLCI	ULCI
General self-efficacy	Perceived task value in sport	0.184	0.034	20.015^***^	0.184	4.474	0.103	0.265
Action planning	Perceived task value in sport	0.371	0.138	45.528^***^	0.185	4.671	0.107	0.263
	General self-efficacy				0.290	7.318	0.212	0.368
Exercise adherence	Perceived task value in sport	0.473	0.224	54.603^***^	0.296	7.733	0.221	0.372
	General self-efficacy				0.218	5.538	0.141	0.295
	Action planning				0.150	3.764	0.072	0.228

^*^p < 0.05, ^**^p < 0.01, ^***^p < 0.001.

Coefficients are standardized regression coefficients (β) obtained from the SPSS PROCESS output and were used to test the paths in the serial mediation model (consistent with Figure 2).

Bootstrap mediation analyzes were conducted, and the results are presented in Table 3. The total association between perceived task value in sport and exercise adherence was significant (β = 0.372, BootSE = 0.039, 95% BootCI [0.296, 0.449]), providing support for H1. After general self-efficacy and action planning were incorporated into the model, the direct association between perceived task value in sport and exercise adherence remained significant (β = 0.296, BootSE = 0.038, 95% BootCI [0.221, 0.372]), suggesting partial statistical mediation.

**Table 3:** Test results for the serial mediation effects.

Path	Effect	Boot SE	Boot CI	Proportion of mediation
Perceived task value in sport → General self-efficacy → Exercise adherence	0.040	0.012	[0.019,0.067]	10.77%
Perceived task value in sport → Action planning → Exercise adherence	0.028	0.011	[0.010,0.052]	7.44%
Perceived task value in sport → General self-efficacy → Action Planning → Exercise adherence	0.008	0.003	[0.003,0.016]	2.15%
Total effect	0.372	0.039	[0.296,0.449]	–
Direct effect	0.296	0.038	[0.221,0.372]	79.64%
Total indirect effect	0.076	0.018	[0.044,0.114]	20.36%

Subsequent bootstrap analyses indicated that all specific indirect pathways were significant. (1) Perceived task value in sport → general self-efficacy → exercise adherence: The indirect estimate was 0.040 (BootSE = 0.012, 95% BootCI [0.019, 0.067]), accounting for 10.77% of the total association, providing support for H2. (2) Perceived task value in sport → action planning → exercise adherence: The indirect estimate was 0.028 (BootSE = 0.011, 95% BootCI [0.010, 0.052]), accounting for 7.44% of the total association, providing support for H3. (3) Perceived task value in sport → general self-efficacy → action planning → exercise adherence: The chain indirect estimate was 0.008 (BootSE = 0.003, 95% BootCI [0.003, 0.016]), accounting for 2.15% of the total association, providing support for H4. Overall, the total indirect estimate was 0.076 (BootSE = 0.018, 95% BootCI [0.044, 0.114]).

BootCI denotes the bias-corrected 95% bootstrap confidence interval (5,000 resamples). The percentage of the total effect mediated (%) was calculated as (indirect effect/total effect) × 100%.

## Discussion

The results of this study indicated significant positive associations among adolescents' perceived task value in sport, general self-efficacy, action planning, and exercise adherence. Further analyzes showed that perceived task value in sport was directly associated with exercise adherence and was also linked to exercise adherence through multiple indirect pathways. (1) perceived task value affected exercise adherence via the mediating role of general self-efficacy; (2) perceived task value also affected exercise adherence via the mediating role of action planning; and (3) perceived task value influenced exercise adherence through the chain mediating pathway of general self-efficacy and action planning. Together, these findings delineate a coherent psychological–behavioral transmission process from value endorsement at the cognitive level to adherence in behavioral implementation, clarifying the internal cognitive mechanisms and behavioral change logic underlying adolescents' exercise adherence. This evidence provides empirical support for a deeper understanding of the psychological processes that sustain adolescents' sport participation and, to some extent, enriches theoretical perspectives in the psychology of adolescent physical activity behavior.

### The relationship between perceived task value in sport and exercise adherence

The results of this study showed that adolescents' perceived task value in sport significantly and positively predicted exercise adherence, supporting H1. This finding is consistent with the conclusions reported by Duda et al. (1989) and further supports the core proposition of expectancy–value theory that behavioral persistence is value-driven (Duda et al., 1989; Feather, 1992). According to this framework, individuals' evaluations of a behavior's importance, utility, and enjoyment shape their cost–benefit trade-offs and resource investment, thereby determining the level of effort and persistence (Pfund et al., 2022). Specifically, when adolescents are more convinced that exercise is “worth investing in,” they are more likely to prioritize exercise in their time allocation and to maintain a stronger tendency to persist when facing barriers such as fatigue or academic conflicts. More importantly, adolescents' sport participation is easily disrupted by academic pressure and situational choices, and often lacks stable external constraints; thus, whether they continue exercising depends to a greater extent on their value judgments regarding the importance, enjoyment, and usefulness of exercise, making the role of perceived task value particularly salient (Brown et al., 2023). Taken together, perceived task value in sport can directly promote sustained investment in exercise behavior and also provides a prerequisite for subsequent self-regulatory mechanisms such as self-efficacy and action planning. Therefore, school-based physical education practice may benefit from targeting perceived task value as an entry point—by optimizing course experiences and providing positive feedback—to support the sustained development of adolescents' exercise adherence.

### The mediating role of general self-efficacy

Further analyzes showed that general self-efficacy partially mediated the relationship between perceived task value in sport and exercise adherence, thereby supporting H2. This finding not only aligns with a central proposition of self-efficacy theory—that efficacy beliefs play a critical role in the initiation and maintenance of behavior—but also highlights the importance of efficacy beliefs as a cognitive psychological resource in the development of exercise persistence (Maddux, 2009; García-Calvo et al., 2011). This result is consistent with Li et al. (2025), who suggested that self-efficacy serves as an important psychological bridge linking value appraisals or motivation to exercise adherence; individuals with higher self-efficacy are more likely to persist rather than withdraw when encountering barriers in exercise contexts (Li et al., 2025). In addition, Lent et al. (1984) reported that the association between self-efficacy and persistence is relatively stable across different motivational backgrounds, further underscoring the comparatively independent role of efficacy beliefs in exercise persistence (Lent et al., 1984).

Specifically, when adolescents rate exercise as more important, enjoyable, and useful, they are more likely to invest effort and participate consistently, thereby accumulating more mastery experiences. These successful and positive experiences can then be internalized into more favorable expectations about one's exercise capability, forming a more stable sense of self-efficacy. As self-efficacy increases, individuals facing obstacles such as fatigue, limited time, or academic conflicts tend to adopt more problem-focused coping strategies, which helps maintain higher levels of exercise adherence. This stepwise process delineates a clear psychological pathway through which perceived task value is translated into exercise adherence via self-efficacy. Moreover, whereas prior research has often explained exercise persistence primarily from motivational or affective perspectives, the present study introduces general self-efficacy as a key mediator in the perceived task value–exercise adherence link, thereby extending explanations of the psychological mechanisms underlying adolescents' exercise persistence (García-Calvo et al., 2010, 2012). Accordingly, school-based physical education should not only strengthen value-oriented education about exercise, but also create attainable mastery experiences through tiered goals and progressively challenging tasks, and consolidate efficacy beliefs through timely, specific positive feedback to support the long-term maintenance of exercise adherence.

### The mediating role of action planning

Further analyzes indicated that action planning partially mediated the relationship between perceived task value in sport and exercise adherence, supporting H3. This finding suggests that the higher adolescents' perceived task value in sport, the more likely they are to develop more specific action plans, and that such clear behavioral planning further facilitates the development of exercise adherence. This process reflects a progressive “value–planning–persistence” pattern and delineates a clear pathway through which value cognitions are translated into sustained exercise behavior. The result is consistent with Marentes-Castillo et al. (2022), who highlighted planning as a critical bridge for the maintenance of health behaviors; action planning can narrow the gap between intention and behavior, increasing the likelihood that individuals move from “wanting to do” to “actually doing” (Marentes-Castillo et al., 2022). In addition, Ajzen et al. (2009) reported that transforming general intentions into explicit implementation plans can significantly increase both behavior initiation and persistence rates (Ajzen et al., 2009). These findings converge with the present results in underscoring the pivotal role of planning mechanisms in the formation of exercise persistence.

Moreover, this evidence further supports the HAPA proposition regarding the intention–action phase: value endorsement or behavioral intention alone is insufficient to ensure behavior enactment. By reducing uncertainty during execution and lowering the cost of momentary decision-making, action planning increases the likelihood of behavior initiation and maintenance, thereby strengthening exercise persistence (Renner and Schwarzer, 2009). Practically, school-based physical education should not only enrich activity content, optimize exercise environments, and provide sustained motivational support, but also strengthen training in action planning and provide feedback on plan implementation. Such efforts may help adolescents translate value endorsement into stable exercise behavior. Sustained and regular, plan-based participation is a key step in the development and maintenance of exercise adherence, with important implications for adolescents' physical and psychological health and for fostering active, healthy lifestyles.

### The chain mediating roles of general self-efficacy and action planning

An additional important finding of this study is that general self-efficacy and action planning showed a significant serial mediation pathway between perceived task value in sport and exercise adherence, thereby supporting H4. However, the size of this serial indirect pathway was relatively small compared with the direct association, suggesting that it should be interpreted as a supplementary mechanism within the overall model. This result is similar to the findings of Pfeffer and Strobach (2019), while the present study further clarifies the specific psychological–behavioral transmission pathway through which value cognitions influence adolescents' exercise adherence. Specifically, perceived task value in sport first enhanced adolescents' general self-efficacy; strengthened efficacy beliefs subsequently facilitated the development of clearer and more feasible action plans, and the implementation of these plans ultimately contributed to sustained improvements in exercise adherence. This finding aligns with a core proposition of HAPA's intention–action phase, namely that the long-term maintenance of behavior does not rely solely on value endorsement but requires self-regulatory mechanisms such as self-efficacy and action planning to accomplish the transition from intention formation to stable enactment (Renner and Schwarzer, 2009). It also supports a general principle of behavior change in which cognitive resources precede behavioral execution: efficacy beliefs provide a psychological foundation for plan formulation, whereas planning mechanisms offer an implementation safeguard for behavioral persistence (Abdullah, 2019).

Accordingly, interventions aimed at improving adolescents' exercise adherence should simultaneously target the cultivation of perceived task value in sport, the strengthening of general self-efficacy, and training in action planning, thereby forming an integrated strategy characterized by the synergistic pathway of “value–efficacy–planning.” Nonetheless, it is important to note that the present study primarily explains psychological–behavioral change processes at the individual level. External contextual factors such as peer support, family resources, and the school physical education environment may also play an important role in plan execution and behavioral maintenance. Future research could integrate individual psychological mechanisms with social-contextual variables to develop a more comprehensive theoretical model of exercise adherence and to enhance the contextual fit and generalizability of intervention strategies.

## Conclusion and limitations

### Research conclusions

This study found significant positive associations among adolescents' perceived task value in sport, general self-efficacy, action planning, and exercise adherence. Perceived task value in sport was directly associated with exercise adherence and was also indirectly linked to exercise adherence through the independent mediating roles of general self-efficacy and action planning as well as their serial mediation pathway. These findings elucidate a psychological–behavioral pathway through which task value cognitions are translated into sustained exercise behavior via self-regulatory mechanisms, and they offer theoretical and practical implications for school-based physical education and adolescent physical activity promotion.

### Research limitations

Although this study examined the mechanisms linking adolescents' perceived task value in sport to exercise adherence from the perspectives of the Health Action Process Approach, self-determination theory, and expectancy–value theory, several limitations should be noted:

(1) Cross-sectional design. This study employed a cross-sectional questionnaire design, which can only reveal associations among variables and does not allow for strong causal inference. Future research could use longitudinal designs or experimental/intervention studies to further test causal pathways.
(2) Sample representativeness. Participants were drawn from adolescents in specific regions, which may limit the generalizability of the findings to adolescents from other areas or developmental stages. Future studies could recruit more diverse samples and consider incorporating potential moderators (e.g., social support, family environment) to examine heterogeneity in the mediation pathways.
(3) Potentially more complex relations. Although the chain mediation effect was significant, the relationship between general self-efficacy and action planning may be more complex, potentially involving bidirectional or dynamic processes. This warrants further investigation using more refined research designs.
(4) Control variables. Sex and age were not included as control variables in the current mediation model, as the analysis focused on the theoretically specified relations among perceived task value in sport, general self-efficacy, action planning, and exercise adherence. However, demographic characteristics may be associated with adolescents' exercise adherence and may be related to the strength of the observed pathways. Future studies should incorporate relevant demographic covariates to further examine the robustness and generalizability of the mediation model.

## Data Availability

The raw data supporting the conclusions of this article will be made available by the authors, without undue reservation.
